# A Traditional Chinese Medicine, Maoto, Suppresses Hepatitis B Virus Production

**DOI:** 10.3389/fcimb.2020.581345

**Published:** 2021-01-22

**Authors:** Md. Arifur Rahman, Keiji Ueda, Tomoyuki Honda

**Affiliations:** ^1^ Division of Virology, Department of Microbiology and Immunology, Osaka University Graduate School of Medicine, Osaka, Japan; ^2^ Department of Microbiology, Noakhali Science and Technology University, Noakhali, Bangladesh

**Keywords:** hepatitis B virus, Kampo, maoto, tropomyosin β chain (TPM2), cytoskeleton

## Abstract

Worldwide, millions of people suffer from hepatitis B virus (HBV) infection, putting them at a high risk of death from liver cirrhosis and cancer. Although effective anti-HBV drugs have been developed, current drugs have some limitations, as most of them have a risk of significant side effects. Therefore, the discovery of safe and effective anti-HBV drugs is still needed. Natural compounds are considered sources of novel, safe and effective therapeutics. In this study, we screened a library of Kampos, traditional herbal medicines, for suppression of HBV production. Among them, we found that maoto reduced extracellular HBV DNA but not extracellular HBsAg during HBV infection, suggesting that it suppressed HBV production by interfering with HBV nucleocapsid incorporation into viral particles. Furthermore, we revealed that maoto reduced the expression of a host gene, *Tropomyosin β chain (TPM2)*, whose downregulation also suppressed HBV production, similarly to maoto. Since the safety of maoto has been already confirmed, maoto can be considered a candidate anti-HBV agent if the effect is confirmed *in vivo*. In addition, our findings also suggest TPM2 as a novel molecular target for the development of anti-HBV agents.

## Introduction

Hepatitis B is an infectious disease caused by hepatitis B virus (HBV). Despite the development of an effective vaccine, more than 250 million HBV carriers still exist, and 887,000 deaths occurred in 2015 due to acute and chronic HBV-related diseases, including liver cirrhosis (LC) and hepatocellular carcinoma (HCC) (Dienstag, 2008; Schweitzer et al., 2015; WHO, 2015; Honda and Rahman, 2019). Disease progression and severity mostly depend on multiple viral and host factors (Rahman et al., 2016; Al-Sadeq et al., 2019; Jia et al., 2019; Hossain et al., 2020). Pegylated interferon (PEG-IFN) and nucleotide analog therapy are presently considered ‘standard therapy’ for chronic hepatitis B treatment; however, even in combination, they are not 100% effective, show side effects and are expensive (Sonneveld and Janssen, 2010; Bruix et al., 2019). For example, during the initial stage of PEG-IFN therapy, flu-like syndrome, headache, myalgia, fatigue, and local reactions at the injection site are common among patients, whereas in later stages, mild myelosuppressive symptoms, such as neutropenia and thrombocytopenia, and neuropsychiatric symptoms have been reported (Van Zonneveld et al., 2005; Sonneveld and Janssen, 2010). Therefore, the development of novel safe agents with anti-HBV activity is still expected.

HBV is a small DNA virus with a partially double-stranded 3.2 kb relaxed circular DNA (rcDNA) genome (Honda, 2016). The HBV genome encodes at least five genes, *pre-core*, *HBc*, *Pol*, *HBs*, and *HBx* (Beck and Nassal, 2007; Patient et al., 2007). Hepatitis B e antigen (HBeAg) and hepatitis B surface antigen (HBsAg) are HBV-specific antigens derived from the *pre-core/HBc* and *HBs* genes, respectively. Upon infection, HBV repairs the rcDNA genome and forms covalently closed circular DNA (cccDNA) that can act as a template for the synthesis of viral transcripts, including pregenomic RNA (pgRNA), in the nucleus (Summers et al., 1975; Seeger and Mason, 2000; Beck and Nassal, 2007; Li et al., 2017). pgRNA is encapsidated in the cytoplasm, and reverse transcription of pgRNA occurs inside the nucleocapsid to generate progeny rcDNA (Beck and Nassal, 2007). Subsequently, interactions of the nucleocapsid with HBs and/or host proteins trigger envelopment of the nucleocapsid, followed by budding of the viral particles (Ning et al., 2011; Cui et al., 2013; Selzer and Zlotnick, 2015). Only mature, rcDNA-containing nucleocapsids interact with HBs to undergo subsequent envelopment (Cui et al., 2013). The accumulation of double-stranded DNA (dsDNA) as a result of second strand elongation during reverse transcription triggers a structural change in the nucleocapsid, which, in turn, signals to initiate envelopment and secretion of HBV (Perlman et al., 2005; Roseman et al., 2005). Along with infectious HBV particles (Dane particles), a number of viral genome-free subviral particles (SVPs) are also released from infected cells (Mabit and Schaller, 2000). All of these HBV infection steps can be a target of novel anti-HBV therapies.

Throughout human history, plants have been used as a source of medicine for human diseases. Traditional Chinese herbal medicine, also called Kampo, has been used widely for more than 2,000 years to treat various types of diseases, including chronic hepatitis B and showed very little side effect (Liu et al., 2001). Here, we screened a Kampo library for anti-HBV activity and found an effective candidate with anti-HBV activity.

## Materials and Methods

### Kampos

The INM Kampo library was provided by the Institute of Natural Medicine, Toyama University, Toyama, Japan. The library consisted of 42 extracts which were prepared based on Kampo formula. The composition of each Kampo formula is available at TradMPD database (https://dentomed.toyama-wakan.net/index_en) at Toyama University. A mixture of crude drugs (2-times amount of the daily dose) was boiled with 500 ml of purified water for 17 min, followed by heating for 13 min. The decoction was filtrated and freeze-dried to yield dry extract powder. The extracts were dissolved in ultra-pure water at a concentration of 10 mg/ml and kept at −80°C before use.

### Cell Culture

HepG2 cells, a human hepatoma cell line, were cultured with Dulbecco’s modified Eagle’s/Ham’s F-12 base medium (DMEM/Ham’s F-12) supplemented with 10% fetal bovine serum (FBS). HepAD38.7 cells derived from HepG2 cells were maintained with DMEM/Ham’s F-12 supplemented with 10% FBS, 5 µg/ml insulin, and 0.5 mg/ml G418 (Nacalai Tesque, Kyoto, Japan) with 0.4 µg/ml tetracycline (Tet (+)) or without tetracycline (Tet (-)) (Ladner et al., 1997; Ogura et al., 2014). Freshly cultured primary human hepatocyte cells (PXB-cells, PhenixBio, Japan) were cultured with Williams’ Medium E (Gibco) supplemented with 10% FBS, 1% antibiotics-antimycotics, 50 μM hydrocortisone, 3 μg/ml insulin, 5 μg/ml transferrin, 10 ng/ml EGF, 5 ng/ml sodium selenite, and 2 mM L-glutamine. HepG2-NTCP cells are HepG2 cells stably expressing sodium taurocholate cotransporting polypeptide (NTCP), a functional HBV receptor (Yan et al., 2012; Iwamoto et al., 2014). HepG2-NTCP cells were maintained with the same medium used for PXB-cells in addition to 0.5 mg/ml of G418.

### Cell Viability Assay

HepAD38.7 and HepG2-NTCP cell viability was determined by using a CellTiter-Glo Luminescent Cell Viability Assay (Promega, Madison, WI) according to the manufacturer’s instructions. Cells were seeded at a density of 1×10^4^ cells/well in 96-well plates and cultured for 9 days of Kampo treatment. Luminescence of cell lysates was measured using a GloMax Discover microplate reader (Promega).

### Hepatitis B Virus Preparation and Infection

HepAD38.7 is a specialized cell line that produces HBV in the absence of tetracycline (Ogura et al., 2014). The cells were cultured without tetracycline, and the culture supernatant was collected every 3 days for up to 60 days. The culture supernatants were filtered through a 0.22 µm filter, and viruses were purified by polyethylene glycol (PEG) precipitation. The virus was used to infect HepG2-NTCP cells and PXB-cells at 2 × 10^3^ genome equivalents (GEq)/cell. Infection was performed in the presence of 2% DMSO and 4% PEG 8000. After 24 h of infection, the cells were washed three times to remove remaining extracellular HBV particles.

### Enzyme-Linked Immunosorbent Assay

The culture supernatant was collected at 9 days post-treatment with Kampos, and HBeAg and HBsAg in the supernatant were evaluated by an HBeAg Diagnostic Kit (Shanghai Rongsheng Biotech Co., Ltd, China) and an HBs S Antigen Quantitative ELISA Kit, Rapid-II (Beacle, Inc, Kyoto, Japan), respectively, according to the manufacturers’ instructions. The luminescence signal was measured using a SpectraMax 190 Microplate Reader (Molecular Devices, Sunnyvale, CA).

### Evaluation of Hepatitis B Virus DNA

HBV extracellular DNA and core-associated DNA (core-DNA) were isolated as described previously with minor modifications (Kleines et al., 2003; Gao and Hu, 2007; Cui et al., 2013). Briefly, the culture supernatant was treated with DNase at 37°C for 30 min, and extracellular DNA was extracted by a QIAmp DNA Mini Kit (Qiagen, Hilden, Germany) according to the manufacturer’s protocol. For isolation of core-DNA, the cells were lysed in lysis buffer (50 mM Tris-HCl [pH 8.0], 1 mM EDTA, 1% Nonidet P-40) (Gao and Hu, 2007). After removal of the nuclear pellet by centrifugation, the supernatant was treated with 20 U/ml DNase (Takara, Shiga, Japan), 5 µg/ml RNase (Roche Diagnostics GmbH, Mannheim, Germany), 5 mM MgCl_2_ and 5 mM CaCl_2_ at 37°C for 3 h to degrade the nucleic acids outside the nucleocapsids. The DNase was then inactivated by the addition of 10 mM EDTA. After the inactivation of DNase, proteinase K (final concentration, 0.6 mg/ml), sodium dodecyl sulfate (SDS; 0.5%) and NaCl (50 mM) were added to disrupt the nucleocapsids. Finally, core-DNA was isolated by a QIAmp DNA Mini Kit (Qiagen). The amounts of extracellular DNA and core-DNA were measured by real-time PCR using Fast SYBR Green Mater Mix (Applied Biosystems) with the HBs-specific primers. The thermal profile was as follows: 40 cycles of 95°C for 1 s and 60°C for 20 s.

### Cesium Chloride Density Gradient Centrifugation

HBV enveloped particles (Dane particles) were purified by equilibrium centrifugation in cesium chloride (CsCl) density gradients. Briefly, 500 µl of culture supernatant was layered onto CsCl gradients and centrifuged for 20 h in a SW40 rotor (Beckman Coulter) at 50,000 rpm. The resultant fractions (150 µl each) were collected from the top of the tube. The fractions containing both HBsAg and HBV DNA were considered as Dane particle fractions.

### Transcriptomic Analysis

Total RNA was isolated from maoto-treated and untreated HepAD38.7 cells and subjected to RNA-seq analysis. Library preparation was performed using a TruSeq stranded mRNA sample prep kit (Illumina, San Diego, CA) according to the manufacturer’s instructions. Sequencing was performed on an Illumina NovaSeq 6000 platform. Sequenced reads were mapped to the human reference genome sequences (hg38) using HISAT2. The fragments per kilobase of exon per million mapped fragments (FPKMs) were calculated using Cufflinks. The expression of cellular mRNAs was then compared between the cells with and without maoto treatment. Differentially expressed genes were identified by selecting genes that were more than 20-fold downregulated compared to the untreated control. We set the cut-off FPKM value as 0.1 for genes in untreated cells. Of these downregulated genes, we further selected genes localized in the cytoplasm. The data have been deposited with links to BioProject accession number **PRJDB10116** in the DDBJ BioProject database.

### Gene Expression Analysis

Gene expression was analyzed as described previously (Nishikawa et al., 2018; Nakayama et al., 2019). Briefly, total RNA was extracted from cells using TRIzol reagent (Thermo Fisher Scientific, Waltham, MA) according to the manufacturer’s instructions. Reverse transcription (RT) of the extracted RNA was carried out using a Verso cDNA Synthesis Kit (Thermo Fisher Scientific). Gene expression was then evaluated using Fast SYBR Green Mater Mix with gene-specific primers in a QuantStudio 6 Flex real-time PCR system (Applied Biosystems). The thermal profile was as follows: 40 cycles of 95°C for 1 s and 60°C for 20 s.

### Plasmid Preparation

To generate plasmids expressing short hairpin RNA (shRNA) against TPM2 (sh1-TPM2 and sh2-TPM2), a pair of oligos (5’-ACC GGC AGA GAA ATC TGC ATT CTA TTG TTA ATA TTC ATA GCA ATA GAA TGC AGA TTT CTC TGT TTT-3’ and 5’-CGA AAA AAC AGA GAA ATC TGC ATT CTA TTG CTA TGA ATA TTA ACA ATA GAA TGC AGA TTT CTC TGC-3’ for sh1-TPM2; 5’- ACC GGG TAT TCT GAA TCT GTG AAG GAG TTA ATA TTC ATA GCT CCT TCA CGG ATT CAG AAT ACT TTT-3’ and 5’CGA AAA AAG TAT TCT GAA TCC GTG AAG GAG CTA TGA ATA TTA ACT CCT TCA CAG ATT CAG AAT ACC-3’ for sh2-TPM2) were annealed and inserted into the *Bbs*I sites of pRSI9-U6-(sh)-UbiC-RFP-2A-Puro (Cellecta). To generate an HBV-expressing plasmid (pHBI), a 1.5-fold HBV genome (accession number **X01587**) (Fujiyama et al., 1983) was cloned into pBR322 (Takara). The full-length TPM2 transcript (accession number **XM_017015092**) was cloned into pLV-SIN-CMV-Hyg (Takara).

### Transient Hepatitis B Virus Production

HepG2 cells were cotransfected with pHBI together with pGL3 (Promega) to normalize the transfection efficiency and an shRNA-expressing plasmid using GenJet *In Vitro* DNA Transfection Reagent (Ver. II) (SignaGen Laboratories, Ijamsville, MD) according to the manufacturer’s instructions. At 7 days post-transfection, the culture supernatant was used to measure the level of HBV production by real-time PCR, and cell lysates were used to check the transfection efficiency by a Luciferase Assay System (Promega).

### Establishment of Tropomyosin β Chain-Overexpressing Cells

Lentivirus containing the TPM2 transgene was generated using pLV-SIN-CMV-Hyg-TPM2. HepG2-NTCP cells were infected with the resultant lentivirus. The cells were then treated with hygromycin B (Wako Pure Chemicals, Tokyo, Japan) at 1 mg/mL to select the hygromycin B resistant clones that ectopically expressed TPM2.

### Western Blot

Western blot was conducted as described previously (Nakayama et al., 2019; Teng et al., 2019). Briefly, the cell homogenate was subjected to SDS-PAGE and transferred onto polyvinylidene difluoride membranes. The membranes were then blocked with 5% skim milk and incubated with an antibody to TPM2 (GeneTex, Irvine, CA) or Tubulin (Wako Pure Chemicals). After three washes with 0.05% Tween 20 in TBS, the membranes were incubated with horseradish peroxidase-conjugated secondary antibodies for 1 h at 37 °C. The bound antibodies were detected using a Clarity Western ECL Substrate (BioRad).

### Immunofluorescence Assay

Immunofluorescence assay was performed as described previously (Nakayama et al., 2019). Briefly, the cells were fixed for 30 min in 4% paraformaldehyde and permeabilized by incubation in PBS containing 0.25% Triton X-100 for 10  min. After permeabilization, the cells were incubated with an antibody to TPM2 for 2 h, followed by incubation with the appropriate Alexa Fluor-conjugated secondary antibodies. The cells were counterstained with 4’,6-diamidino-2-phenylindole (DAPI). A confocal laser-scanning microscope Leica TCS SP8 HyVolution (Leica Microsystems) was used for cell immunofluorescence imaging and data collection.

### Primers

The primers and probes used in this study were as follows:

HBsF_2_: 5′-CTT CAT CCT GCT GCT ATG CCT-3′
HBsR_2_: 5′-AAA GCC CAG GAT GAT GGG AT-3′
GAPDH mRNA-F: 5′-AGC GAG ATC CCT CCA AAA TC-3′
GAPDH mRNA-R: 5′-AAA TGA GCC CCA GCC TTC TC-3′
TPM2 mRNA-F: 5′-GTG GGG ACC TAG AGG AGG AG-3′
TPM2 mRNA-R: 5′-CAC AGA CCT CTC GGC AAA CT-3′

### Statistical Analysis

Statistical significance was assessed using a two-tailed Student’s *t*-test with a threshold of *P* < 0.05.

## Results

### Screening of a Kampo Library for Suppression of Hepatitis B Virus Production

**Figure 1:**
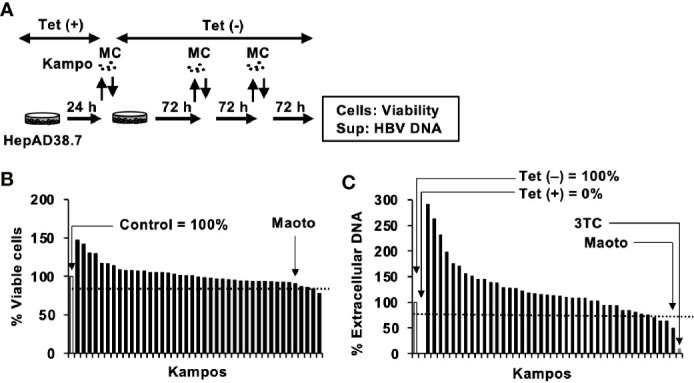
Screening of Kampos for anti-hepatitis B virus (HBV) activity in HepAD38.7 cells. **(A)** Schematic representation of the screening protocol. Cells were treated with distilled water (control) or 100 µg/ml Kampo extract in the culture medium without tetracycline (Tet(-)) to induce HBV production. The cells were incubated for 9 days with refreshing the medium and Kampo (MC) every 3 days. **(B)** Viability of HepAD38.7 cells. Cells were treated with 100 µg/ml Kampo for 9 days with refreshing the medium and Kampo every 3 days. Viability was determined after 9 days of treatment using the CellTiter-Glo assay. **(C)** Extracellular HBV DNA. HBV DNA in culture supernatants of HepAD38.7 cells was measured by real-time PCR. Lamivudine (1 µM, 3TC) was used as a positive control for the inhibition of HBV production. Values of the primary screening are shown.

To identify anti-HBV Kampos, the INM Kampo library, containing 42 Kampo extracts, was first checked for cell toxicity. HepAD38.7 cells were treated with Kampo extracts, and cell viability was evaluated after 9 days of Kampo treatment (**Figure 1A**). Among the 42 extracts, 41 resulted in >80% viability (**Figure 1B**). Then, the extracts without cytotoxicity were screened for their anti-HBV activity. HBV production was induced by removing tetracycline, and simultaneously, the cells were treated with or without Kampo extracts to evaluate the anti-HBV activity. Four of the 41 extracts suppressed extracellular HBV production in HepAD38.7 cells by >25% (**Figures 1A, C**). Since the maoto extract suppressed HBV production the most (**Figure 1C**), we focused on the maoto extract for further investigation.

### Maoto Suppresses Extracellular Hepatitis B Virus Production in HepAD38.7 Cells

**Figure 2:**
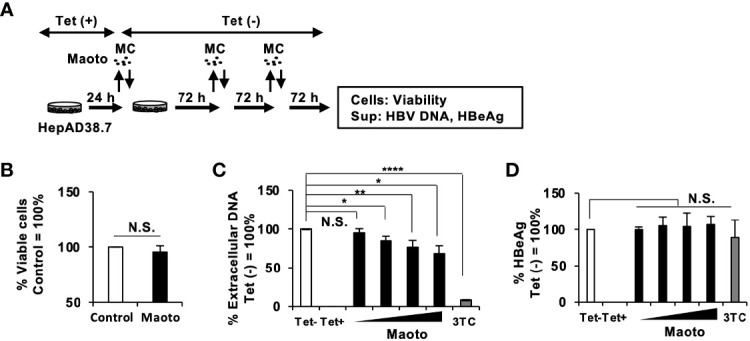
Maoto dose-dependently inhibits hepatitis B virus (HBV) production in HepAD38.7 cells. **(A)** Schematic representation of the protocol of maoto treatment in HepAD38.7 cells. Cells were treated with distilled water (control) or concentrations of 1.5625, 6.25, 25, and 100 µg/ml of maoto extract in the culture medium without tetracycline (Tet(-)) to induce hepatitis B virus (HBV production. The cells were incubated for 9 days with refreshing the medium and Kampo (MC) every 3 days. **(B)** Cell viability in HepAD38.7 cells. Cells were treated with 100 µg/ml maoto extract for 9 days. Viability was determined after 9 days of treatment using the CellTiter-Glo assay. **(C)** Extracellular HBV DNA. HBV DNA in culture supernatants of HepAD38.7 cells was measured by real-time PCR. **(D)** Extracellular HBeAg. HBeAg in culture supernatants of HepAD38.7 cells was determined by enzyme-linked immunosorbent assay (ELISA). Lamivudine (1 µM, 3TC) was used as a positive control for the inhibition of HBV production. Values are expressed as the mean percentage + S.E. of three independent experiments. *P < 0.05; **P < 0.01; ****P < 0.001; N.S., no significance.

To confirm the effect of maoto, we evaluated it in HepAD38.7 cells in more detail (**Figure 2A**). We first confirmed that the maoto extract did not show any cytotoxicity at a concentration of 100 µg/ml (**Figure 2B**). Then, the effect of maoto on HBV production was evaluated at various concentrations under 100 µg/ml (**Figures 2C, D**). Consistent with the results in **Figure 1C**, maoto inhibited extracellular HBV production in HepAD38.7 cells in a dose-dependent manner (**Figure 2C**). Maoto did not affect HBeAg production, which is thought to correlate with HBV pgRNA production, in the tested concentration range (**Figure 2D**). These results confirmed the inhibitory effect of maoto on extracellular HBV production without any cytotoxicity and suggest that maoto inhibits a step in the HBV production process after HBV pgRNA expression.

### Maoto Suppresses Hepatitis B Virus Production in the Context of Infection

**Figure 3:**
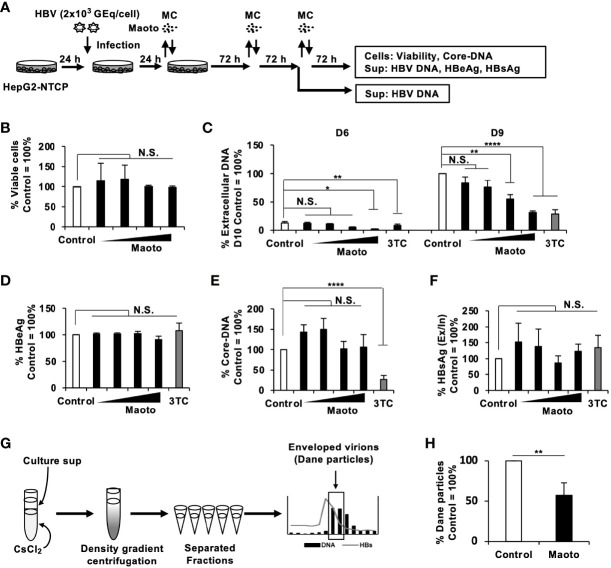
Maoto inhibits hepatitis B virus (HBV) production by interfering with HBV nucleocapsid incorporation into viral particles in HepG2-NTCP cells. **(A)** Schematic representation of the protocol of maoto treatment and HBV infection in HepG2-NTCP cells. Cells were infected with HBV and then treated with distilled water (control) or concentrations of 1.5625, 6.25, 25, and 100 µg/ml of maoto extract for 9 days with refreshing the medium and Kampo (MC) every 3 days. **(B)** Viability of HepG2-NTCP cells. Cells were treated with concentrations of 1.5625, 6.25, 25, and 100 µg/ml of maoto extract for 9 days. Viability was determined after 9 days of treatment using the CellTiter-Glo assay. **(C)** Extracellular HBV DNA. HBV DNA in culture supernatants of the cells after 6 and 9 days of maoto treatment was measured by real-time PCR. **(D)** Extracellular HBeAg. HBeAg in culture supernatants of the cells after 9 days of maoto treatment was determined by ELISA. **(E)** Core-DNA. Core-DNA was extracted from the cells after 9 days of maoto treatment and measured by real-time PCR. **(F)** The Ex/In HBsAg ratio. Extracellular and intracellular HBsAg after 9 days of maoto treatment was determined by enzyme-linked immunosorbent assay (ELISA), and the ratio was calculated by dividing extracellular HBsAg with intracellular HBsAg. **(G)** Schematic representation of the protocol of Dane particle purification. The fractions containing both HBV DNA and HBsAg were considered enveloped virion (Dane particles). **(H)** Effect of maoto on Dane particle production. The amount of Dane particles was determined by measuring HBV DNA with real-time PCR. Lamivudine (1 µM, 3TC) was used as a positive control for the inhibition of HBV production. Values are expressed as the mean percentage + S.E. of three or four independent experiments. *P < 0.05; **P < 0.01; ****P < 0.001; N.S., no significance.

Next, we examined the relevance of the inhibitory effect of maoto on HBV production in the context of HBV infection using HepG2-NTCP cells and primary human hepatocytes isolated from PXB-mice (PXB-cells). HepG2-NTCP cells and PXB-cells were infected with HBV at 2×10^3^ GEq/cell and treated with maoto at the indicated concentrations (**Figure 3A** and **Figure S1A**). The effects of maoto on HBV were evaluated at 6 and 9 days of maoto treatment in HepG2-NTCP cells and at 11 days of the treatment in PXB-cells (**Figure 3A** and **Figure S1A**). As shown in **Figure 3B** and **Figure S1B**, maoto did not exhibit any cytotoxicity to HepG2-NTCP cells and PXB-cells. We then found that maoto treatment resulted in dose-dependent suppression of extracellular HBV DNA production without affecting HBeAg expression, similar to the effects in HepAD38.7 cells (**Figures 3C, D**, and **Figures S1C, D**). The IC_50_ of the maoto extract to inhibit HBV production was 33.2 ± 5.6 µg/ml in HepG2-NTCP cells and 7.5 ± 5.4 µg/ml in PXB-cells (**Figure 3C** and **Figure S1C**). These results confirmed the relevance of the inhibitory effect of maoto on HBV in the context of HBV infection.

### Maoto Interferes With Hepatitis B Virus Nucleocapsid Incorporation Into Viral Particles

To understand the mechanisms of action of maoto, we next evaluated the amount of intracellular core-DNA, which is a reverse-transcribed product of pgRNA, in HBV-infected HepG2-NTCP cells and PXB-cells. Although extracellular HBV production was suppressed by maoto (**Figure 3C** and **Figure S1C**), we did not detect any reduction in core-DNA by maoto treatment (**Figure 3E** and **Figure S1E**). We therefore reasoned that HBV budding might be decreased by maoto treatment. To evaluate this possibility, we determined the ratio of the amount of extracellular HBsAg to that of intracellular HBsAg (the Ex/In HBsAg ratio) during maoto treatment. In contrast to our hypothesis, the Ex/In HBsAg ratio in maoto-treated cells was comparable to that in control cells (**Figure 3F** and **Figure S1F**), suggesting that HBV budding process seemed not affected by maoto treatment. We then examined if production of HBV enveloped particles containing HBV DNA (Dane particle) was decreased by maoto treatment. We purified Dane particles using CsCl density gradient centrifugation as shown in **Figure 3G** and found that the amount of HBV Dane particles was decreased by maoto treatment (**Figure 3H**). Taken together, these results suggest that maoto likely interferes with the process of HBV nucleocapsid incorporation into viral particles.

### Combined Use of Maoto and Lamivudine Decreases Hepatitis B Virus Replication More Efficiently Than the Individual Use

**Figure 4:**
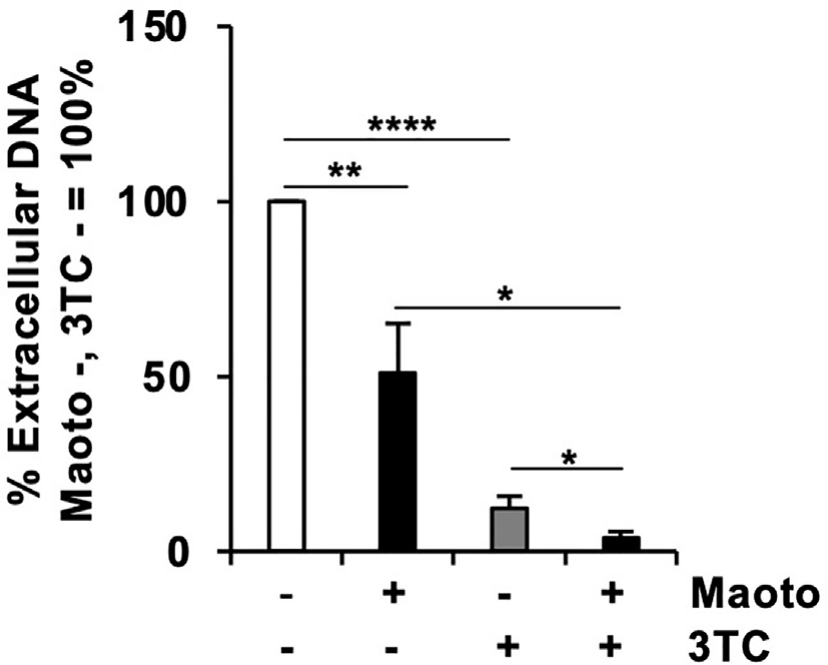
Combined use of maoto and 3TC decreases hepatitis B virus (HBV) replication more efficiently than the individual use. Cells were treated with distilled water (control), 30 µg/ml maoto alone, 250 nM lamivudine (3TC) alone, or maoto and 3TC for 9 days with refreshing the medium and drugs every 3 days as shown in **Figure 3A**. HBV DNA in culture supernatants of the cells after 9 days of maoto treatment was measured by real-time PCR. Values are expressed as the mean percentage + S.E. of three independent experiments. *P < 0.05; **P < 0.01; ****P < 0.001; N.S., no significance.

Considering that maoto suppresses an HBV infection step different from that of lamivudine (3TC), i.e., the reverse transcription step, we reasoned that a combined use of 3TC and maoto would suppress HBV replication more effectively. We therefore compared the effect of maoto alone, 3TC alone, and the combined use on HBV replication. Compared to maoto or 3TC alone, the combined use decreased HBV replication more efficiently as expected (**Figure 4**). These results suggest the potential of maoto for increasing the efficacy of current anti-HBV drugs when used in combination.

### Tropomyosin β Chain Supports Extracellular Hepatitis B Virus Production

**Figure 5:**
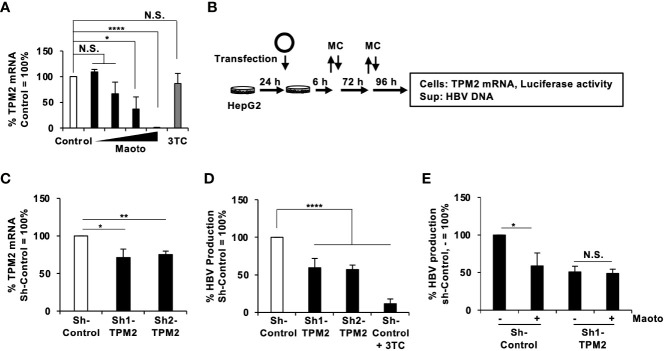
Tropomyosin β chain (TPM2) knockdown decreases hepatitis B virus (HBV) production. **(A)** TPM2 mRNA in maoto-treated cells. HepG2-NTCP cells were treated with distilled water (control) or concentrations of 1.5625, 6.25, 25, and 100 µg/ml of maoto extract for 9 days. The amount of TPM2 mRNA was measured by real-time RT-PCR. **(B)** Schematic representation of the protocol of maoto treatment and transfection of an HBV-expressing plasmid together with an sh-TPM2-expressing plasmid in HepG2 cells. The remaining plasmids were washed at 6 h after the transfection and the medium was refreshed (MC). The medium was further refreshed at 3 days after the transfection (MC). **(C)** TPM2 mRNA in sh-TPM2-expressing cells. Knockdown of TPM2 was confirmed at 7 days after the transfection by real-time RT-PCR. **(D)** Effect of TPM2 knockdown on HBV production. HBV DNA in culture supernatants of the cells was measured by real-time PCR and normalized with the transfection efficiency based on luciferase activity of the cells. Lamivudine (1 µM, 3TC) was used as a positive control for the inhibition of HBV production. **(E)** Effect of maoto extract on HBV production in sh-TPM2-expressing cells. Values are expressed as the mean percentage + S.E. of three independent experiments. *P < 0.05; **P < 0.01; ****P < 0.001; N.S., no significance.

**Figure 6:**
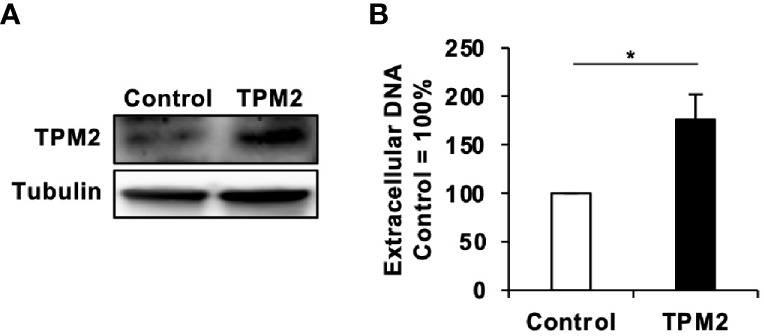
Tropomyosin β chain (TPM2) overexpression increases HBV production. **(A)** TPM2 expression in TPM2-expressing HepG2-NTCP cells. Overexpression of TPM2 in TPM2-expressing HepG2-NTCP cells was confirmed by western blot. Tubulin was used as an internal control. **(B)** Extracellular HBV DNA. TPM2-expressing HepG2-NTCP cells were infected with HBV and then cultured for 9 days with refreshing the medium every 3 days. HBV DNA in culture supernatants of the cells was measured by real-time PCR. Values are expressed as the mean percentage + S.E. of three independent experiments. *P < 0.05.

Finally, we investigated the molecular mechanisms of how maoto suppressed extracellular HBV production. To this end, we analyzed the transcriptomes of maoto-treated and untreated cells. RNA-seq analysis of total RNA extracted from maoto-treated cells revealed that 38 genes were downregulated by maoto treatment by >20-fold. Because maoto likely suppressed the process of HBV nucleocapsid incorporation into HBV particles, which occurs in the cytoplasm, we focused on the cytoplasmic proteins, which consisted of 6 downregulated gene products. Among the six genes (*TPM2*, *TNNT2*, *MYO18B*, *CYP2D7*, *SEC1B*, *GSTT2B*), *Tropomyosin β chain* (*TPM2*) was most strongly downregulated by maoto; therefore, we focused on this gene for further analyses. By real-time RT-PCR analysis, we confirmed that TPM2 was dose-dependently downregulated by maoto treatment (**Figure 5A**). We then examined whether a reduction in TPM2 expression decreases HBV production, as found in the maoto treatment. We transfected an HBV-expressing plasmid, pHBI, and an sh-TPM2-expressing plasmid and evaluated HBV production (**Figure 5B**). Downregulation of TPM2 expression by sh-TPM2 was confirmed by measuring the amount of TPM2 mRNA (**Figure 5C**) as well as by immunofluorescence assay (**Figure S2**). In this setting, TPM2 knockdown suppressed HBV production in the cells transfected with pHBI (**Figure 5D**). Furthermore, the maoto extract did not further decrease HBV production in sh-TPM2-knockdown cells (**Figure 5E**), suggesting that maoto exerted its inhibitory effect through downregulating TPM2 expression. To confirm these results, we evaluated the effect of TPM2 overexpression on extracellular HBV production. Consistent with the results of TPM2 knockdown, TPM2 overexpression increased HBV production (**Figure 6** and **Figure S2**). These results indicate that maoto suppresses HBV production by reducing TPM2 expression.

## Discussion

We screened a library of Kampo extracts for anti-HBV activity in HepAD38.7 cells and found that the maoto extract had anti-HBV activity (**Figures 1** and **2**). Maoto (ma-huang-tang in Chinese), which was developed in ancient China, is used to alleviate flu symptoms such as headache, chill, fever, arthralgia, and cough, and is prescribed as an alternative to neuraminidase inhibitors (drugs commonly used for influenza virus infection) for over 40 years in Japan (Arita et al., 2016; Yoshino et al., 2019). It can also be applied for rheumatoid arthritis, bronchial asthma, infant nasal obstruction, and difficulties in sucking milk in children (Arita et al., 2016; Yoshino et al., 2019). Maoto is a multicomponent formulation originally extracted from four plant products, i.e., ephedra herb (32.3%), apricot kernel (32.3%), cinnamon bark (25.8%), and glycyrrhiza root (9.6%) (Yoshino et al., 2019). It is one of the extensively studied herbal medicines and has been included in national health insurance scheme in Japan. Moreover, it is formulated by following the good manufacturing practice (GMP) stipulated by Japanese law and GMP guidelines to ensure the reproducibility and standardization of biological efficacy as well as for rigorous scientific investigation (Kubo and Nishimura, 2007; Toriumi et al., 2012; Nishi et al., 2017). It has been reported that administration of 180 mg/kg/day (equivalent to ~1200-1350 µg/ml/day) of maoto in children aged between 5 and 10 years old, potentially shortened the duration of fever caused by influenzae A virus without any drug-related adverse effects (Toriumi et al., 2012). In healthy adults between 20 and 45 years of age, maoto has been used at a concentration of 7.5g/day (equivalent to ~1,500 µg/ml/day) (Kitagawa et al., 2019). In our study, we used the maximum concentration of 100 µg/ml of maoto and is much lower concentration than the safety doses based on these reports. Moreover, the drug cost of maoto is 1.4 USD per person/treatment, which is much less than the cost of the alternative treatments (Arita et al., 2016). Together with our findings, these facts indicate that maoto will be a safe and cheap option for an agent with anti-HBV activity although further *in vivo* evaluation is needed.

At present, due to the presence of large and diverse compounds in maoto, it is unclear which compounds of maoto affect the HBV replication. A comprehensive pharmacological profile showed that in total, 352 chemical composition-determined compounds (CCDs) and 113 CCDs were detected in maoto extract and at rat plasma after maoto treatment, respectively (Nishi et al., 2017). Among the 113 CCDs at rat plasma, only 19 were present in maoto extract, while other 94 CCDs were presumed to be generated either from maoto metabolism or endogenous substances produced due to the effects of maoto treatment (Nishi et al., 2017). They also suggested that combination of constituent herbs seems required to exert the effect of maoto because no individual herb could show the effect (Nishi et al., 2017). We also screened all individual herb constituents of maoto; however, we did not find any individual herb that suppressed HBV replication comparable to maoto (data not shown), suggesting that combination of the herb extracts seems required to exert the inhibitory effect on HBV replication.

In this study, we determined the step in the HBV lifecycle targeted by maoto to be HBV nucleocapsid incorporation into HBV particles using HepG2-NTCP cells and PXB-cells (**Figure 3** and **Figure S1**). To date, most anti-HBV drugs target cccDNA, viral transcripts, nucleocapsid assembly, reverse transcription, and the secretion of viral envelope proteins (Durantel and Zoulim, 2016). Maoto reportedly regulates endosomal pH by a vacuolar-type H^+^ ATPase (V-ATPase), inhibiting the uncoating of influenza viruses during the entry (Masui et al., 2017). In our study, however, we used HepAD38.7 cells, where we could only evaluate the HBV processes after pgRNA synthesis, and added maoto after HBV infection in HepG2-NTCP cells and PXB-cells, thus excluding the possibility of HBV entry inhibition. Overall, maoto seems to be a unique candidate that targets a process different from those targeted by existing drugs and can enhance the anti-HBV activity of current drugs when used in combination as shown in **Figure 4**.

Knowledge regarding host factors involved in HBV nucleocapsid envelopment is limited. Several studies have demonstrated that HBc phosphorylation and dephosphorylation events by cellular enzymes occur prior to envelope formation (Mabit and Schaller, 2000). However, we did not find any kinases that were downregulated by maoto treatment. Our transcriptome profiling found that maoto treatment decreased TPM2 expression. Based on the Expression Atlas database (https://www.ebi.ac.uk/gxa/licence.html), TPM2 expression was 2–13 FPKM in liver cells. Consistently, in our RNA-seq, TPM2 expression was ~7.3 FPKM in control cells, whereas it was 0.2 FPKM in maoto-treated cells. Tropomyosins (TPMs) are a family of actin filament-binding cytoplasmic proteins that regulate various cellular functions, including cell motility, adhesion, signaling, vesicle transport and actomyosin contractility (Gunning et al., 2015). A previous study demonstrated that TPM2 interacts with HIV core proteins and supports HIV replication (Santos et al., 2012). Considering that maoto likely suppresses HBV production by interfering with HBV nucleocapsid incorporation into virions, we can speculate that TPM2 might interact with the HBV nucleocapsid to facilitate viral envelopment. When TPM2 is suppressed either by maoto or by shRNAs against TPM2, HBV nucleocapsids cannot incorporate into HBV particles, thereby suppressing the formation of mature HBV DNA-containing Dane particles as shown in **Figure 3H**. On the other hand, overexpression of TPM2 increased HBV production (**Figure 6**). Several transcriptomic analyses have demonstrated that TPM2 expression is increased approximately 4- to more than 6-fold in HBV-associated HCC or acute liver failure, respectively (Hass et al., 2016; Chen et al., 2018). These observations suggest the possibility that HBV may upregulate TPM2 expression and regulate the actin cytoskeleton for its efficient spread and/or replication in the liver. Additionally, we also found *MYO18B* as another gene downregulated by the maoto treatment. MYO18B is reportedly involved in HCC progression by activating the PI3K/AKT/mTOR signaling pathway (Zhang et al., 2018). Since the PI3K/AKT/mTOR pathway regulates the cytoskeleton, similar to TPM2, downregulation of MYO18B may also affect HBV nucleocapsid incorporation into virions together with TPM2. In addition to the actin cytoskeleton, microtubules are known to play vital roles in the transportation of viral components, assembly, and envelopment of a number of viruses, such as vesicular stomatitis virus, hepatitis C virus, and influenza A virus (Amorim et al., 2011; Akil et al., 2016; Yacovone et al., 2016). Host microtubules also support HBV replication, as HBV permissiveness was impaired upon treatment with the microtubule inhibitor nocodazole through inhibition of capsid formation (Iwamoto et al., 2017). However, we excluded this possibility in our study because core-DNA was not affected by maoto (**Figure 3E** and **Figure S1E**), suggesting that capsid formation was not affected.

In conclusion, maoto suppressed HBV production by interfering with HBV nucleocapsid incorporation into virions, possibly through reduction of TPM2 expression. Since the safety of maoto has been already confirmed, maoto will be an attractive candidate drug with anti-HBV activity although further *in vivo* evaluation is needed. Furthermore, our results also propose TPM2 as a novel therapeutic target for HBV infection.

## Data Availability Statement

The data presented in the study are deposited in the DDBJ BioProject repository, accession number PRJDB10116.

## Author Contributions

MAR, KU, and TH conducted the experiments. MAR and TH analyzed the data and wrote the paper. All authors contributed to the article and approved the submitted version.

## Funding

This study was supported in part by AMED under Grant Numbers JP19fk0310101 and JP20fk0310101, JSPS KAKENHI Grant Numbers 18H02664 and 18K19449, and grants from the Takeda Science Foundation, Senri Life Science Foundation, Kobayashi International Scholarship Foundation (TH).

## Conflict of Interest

The authors declare that the research was conducted in the absence of any commercial or financial relationships that could be construed as a potential conflict of interest.
