# Supplementary material for: A Traditional Chinese Medicine, Maoto, Suppresses Hepatitis B Virus Production
Source: Front Cell Infect Microbiol. 2021 Jan 22;10:581345. doi: 10.3389/fcimb.2020.581345 (PMC7862555; doi:10.3389/fcimb.2020.581345)
Supplement: Supplementary file 1 [file DataSheet_1.pdf]

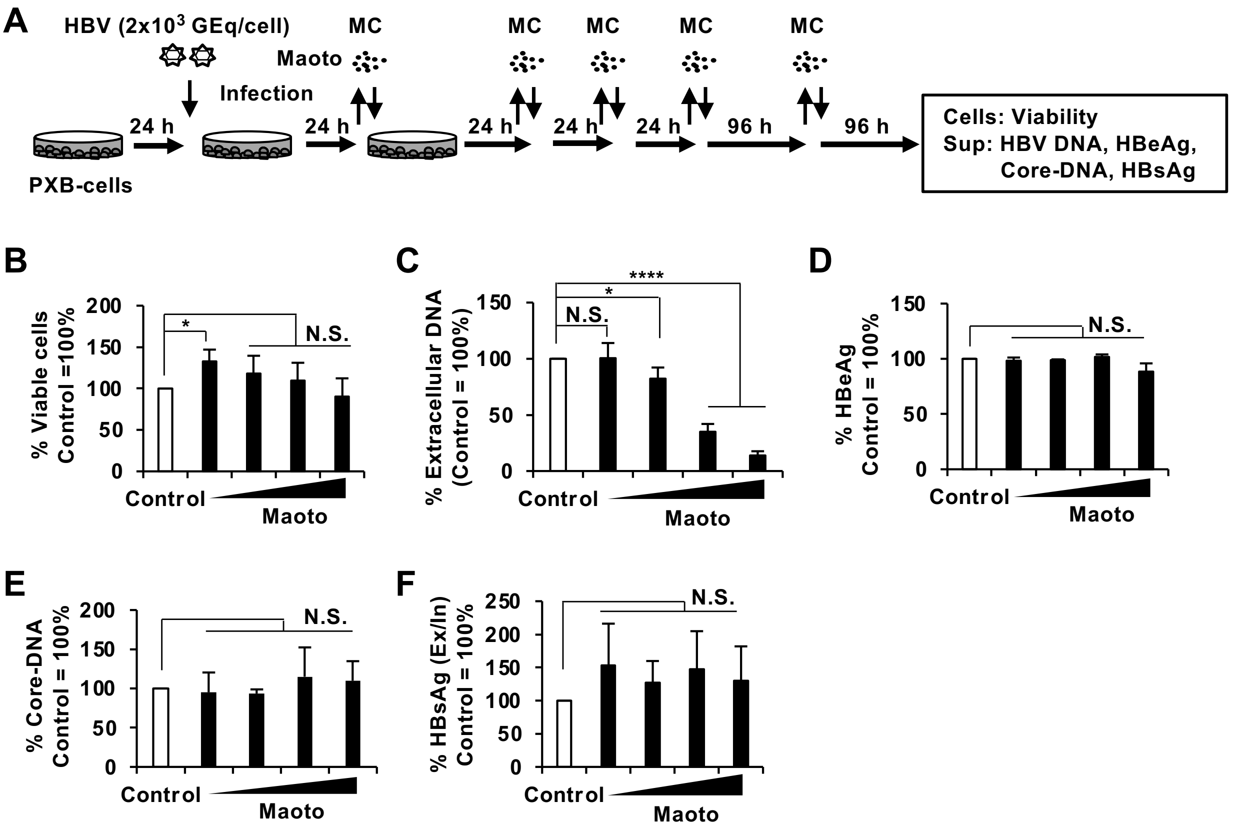

Supplementary Figure S1. Maoto dose-dependently inhibits HBV production in chimeric primary human hepatocytes (PXB-cells). (A) Schematic representation of the protocol of maoto treatment and HBV infection in PXB-cells. Cells were infected with HBV and then treated with distilled water (control) or concentrations of 1.5625, 6.25, 25 and 100 µg/ml of maoto extract for 11 days with refreshing the medium and Kampo at the indicated time points (MC). (B) Viability of PXB-cells. HBV-infected cells were treated with distilled water (control) or concentrations of 1.5625, 6.25, 25 and 100 µg/ml of maoto extract for 11 days. Viability was determined after 11 days of treatment using the CellTiter-Glo assay. (C) Extracellular HBV DNA. HBV DNA in culture supernatants of the cells was measured by real-time PCR. (D) Extracellular HBeAg. HBeAg in culture supernatants of the cells was determined by ELISA. (E) Core-DNA. Core-DNA was extracted from the cells and measured by real-time PCR. (F) The Ex/In HBsAg ratio. Extracellular and intracellular HBsAg was determined by ELISA, and the ratio was calculated by dividing extracellular HBsAg with intracellular HBsAg. Values are expressed as the mean percentage + S.E. of four independent experiments. *, $P < 0.05$; ****, $P < 0.001$; N.S., no significance.

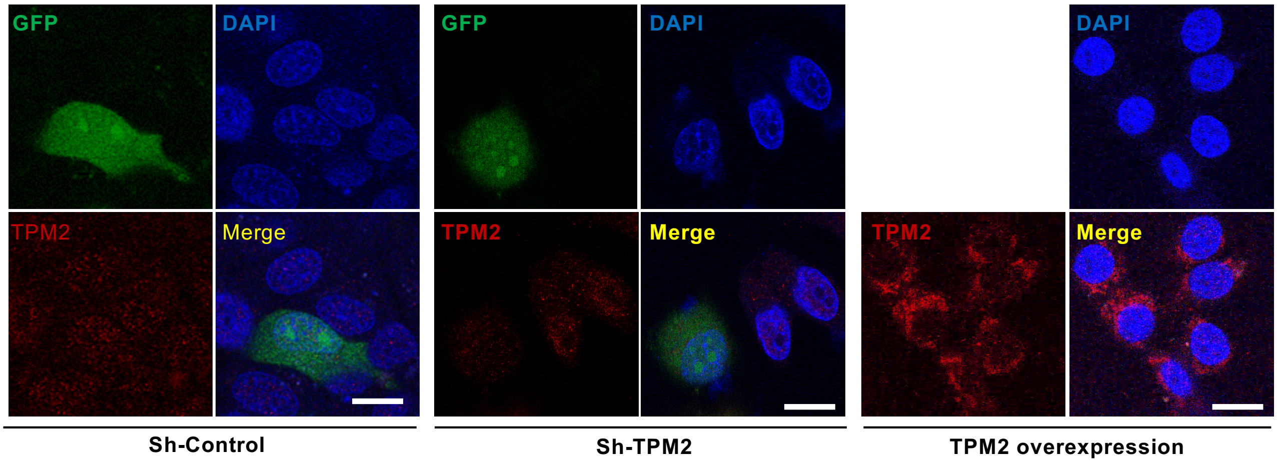

Supplementary Figure S2. TPM2 expression in sh-TPM2- and TPM2-expressing cells. HepG2 cells were transfected with sh-TPM2- and GFP-expressing plasmids. At 48 hours after transfection, the transfected cells and HepG2-NTCP cells stably expressing TPM2 were fixed, permeabilized and stained with a rabbit anti-TPM2 antibody followed by an Alexa Fluor 546-conjugated anti-rabbit IgG (red). The cell nucleus was counterstained with DAPI (Blue). The images are representatives of three independent experiments. GFP-positive cells (green), shRNA-transduced cells. Bars, 10 μm.
